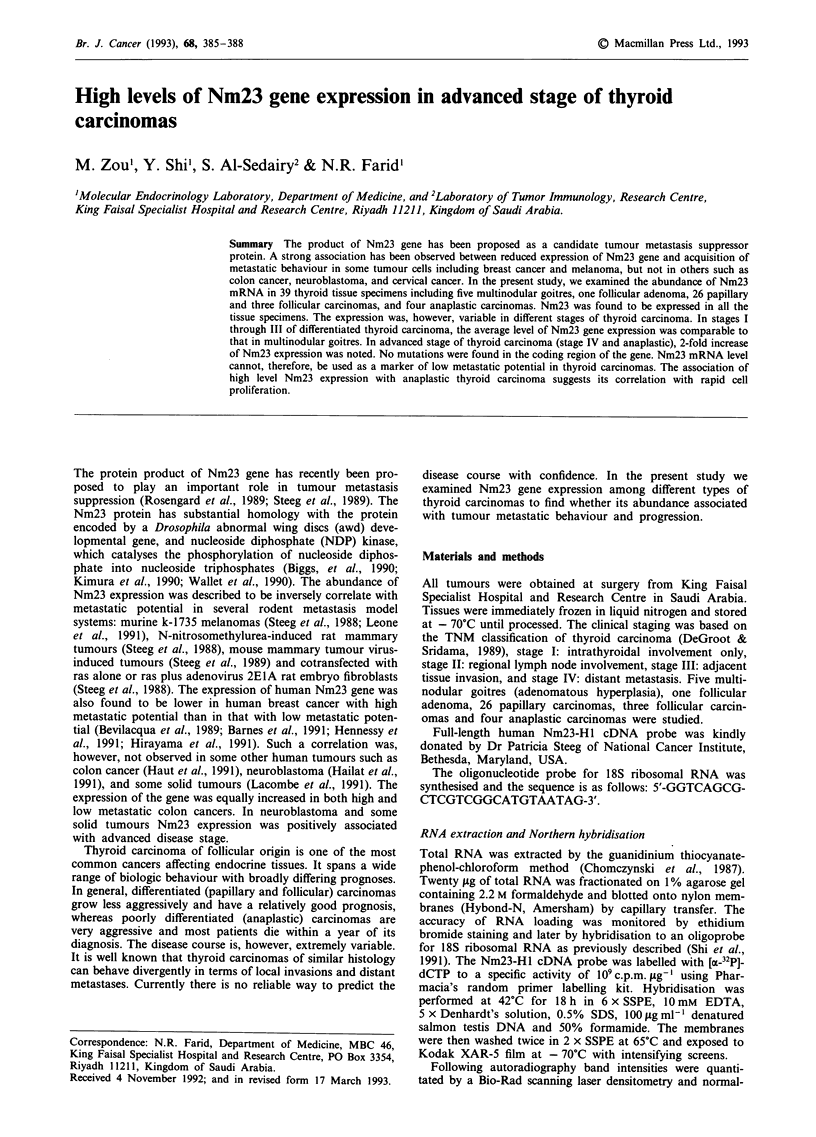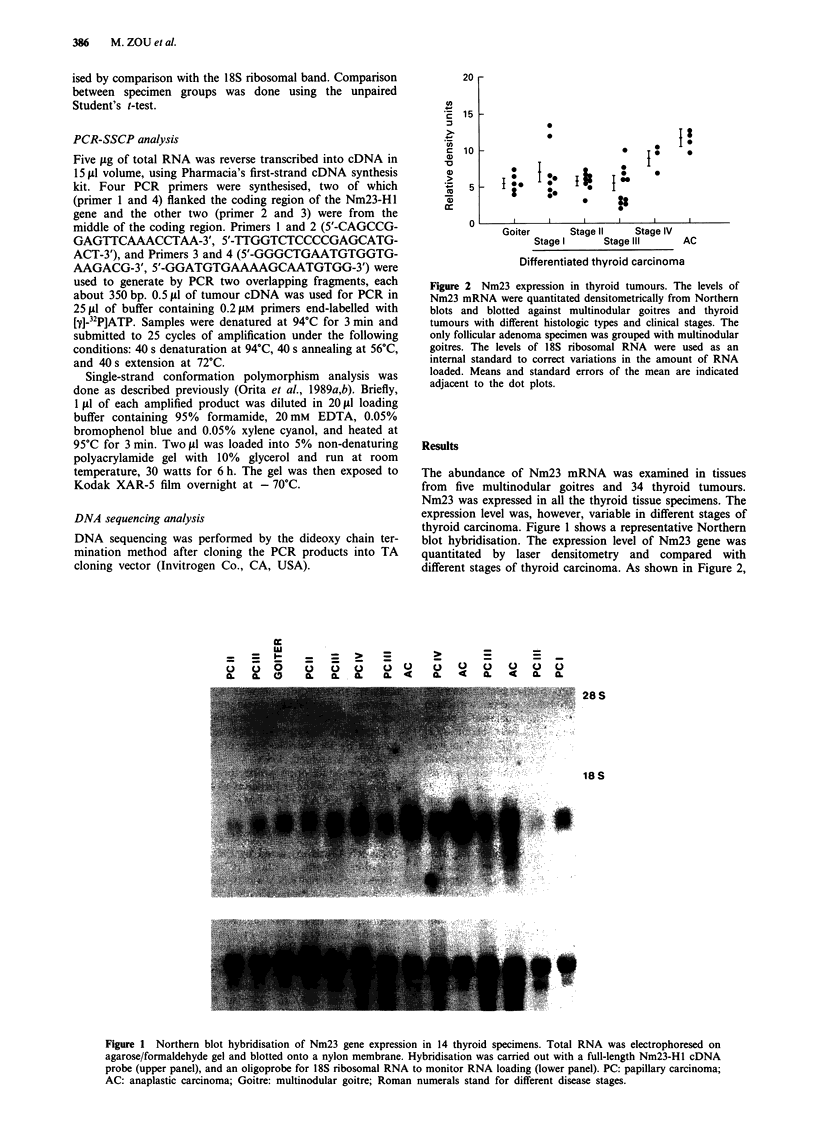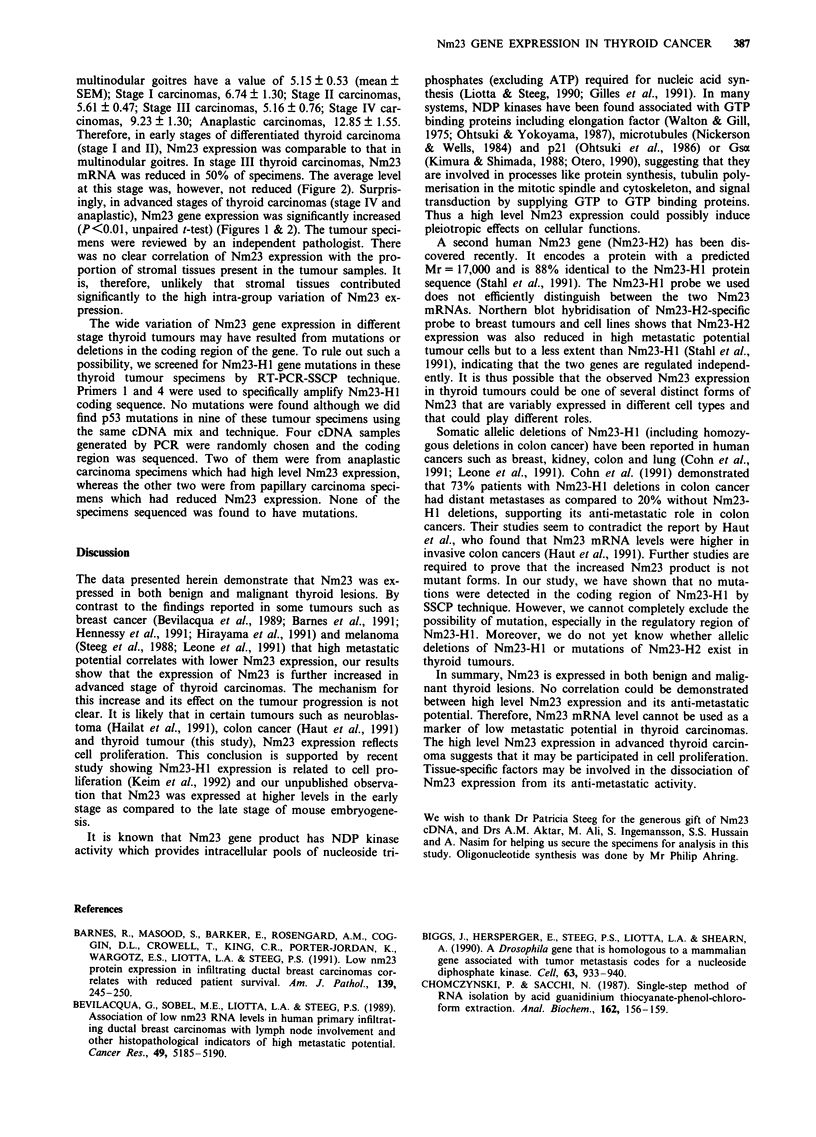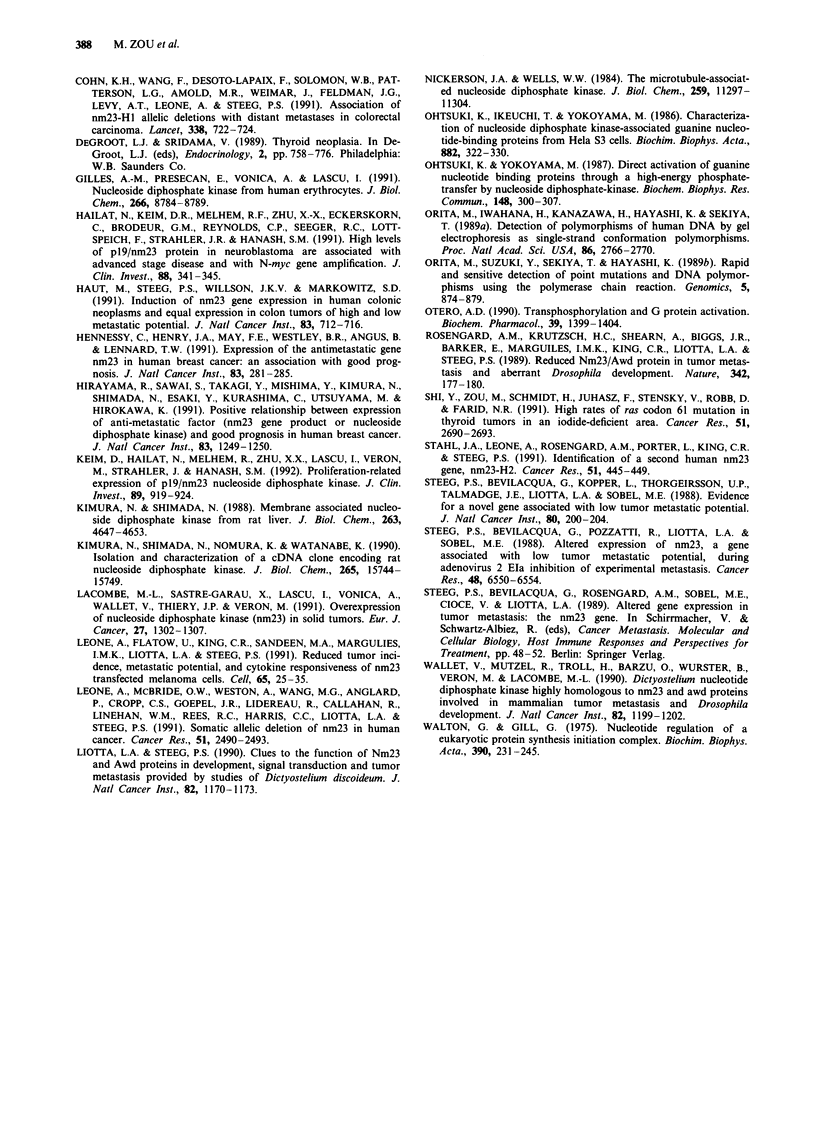# High levels of Nm23 gene expression in advanced stage of thyroid carcinomas.

**DOI:** 10.1038/bjc.1993.345

**Published:** 1993-08

**Authors:** M. Zou, Y. Shi, S. al-Sedairy, N. R. Farid

**Affiliations:** Department of Medicine, King Faisal Specialist Hospital and Research Centre, Riyadh, Kingdom of Saudi Arabia.

## Abstract

**Images:**


Br. J. Cancer (1993), 68, 385-388 © Macmillan Press Ltd., 1993

### High levels of Nm23 gene expression in advanced stage of thyroid carcinomas

M. Zou[1], Y. Shi[1], S. Al-Sedairy[2] & N.R. Farid[1]

[1]*Molecular Endocrinology Laboratory, Department of Medicine, and* [2]*Laboratory of Tumor Immunology, Research Centre, King Faisal Specialist Hospital and Research Centre, Riyadh 11211, Kingdom of Saudi Arabia.*

**Summary** The product of Nm23 gene has been proposed as a candidate tumour metastasis suppressor protein. A strong association has been observed between reduced expression of Nm23 gene and acquisition of metastatic behaviour in some tumour cells including breast cancer and melanoma, but not in others such as colon cancer, neuroblastoma, and cervical cancer. In the present study, we examined the abundance of Nm23 mRNA in 39 thyroid tissue specimens including five multinodular goitres, one follicular adenoma, 26 papillary and three follicular carcinomas, and four anaplastic carcinomas. Nm23 was found to be expressed in all the tissue specimens. The expression was, however, variable in different stages of thyroid carcinoma. In stages I through III of differentiated thyroid carcinoma, the average level of Nm23 gene expression was comparable to that in multinodular goitres. In advanced stage of thyroid carcinoma (stage IV and anaplastic), 2-fold increase of Nm23 expression was noted. No mutations were found in the coding region of the gene. Nm23 mRNA level cannot, therefore, be used as a marker of low metastatic potential in thyroid carcinomas. The association of high level Nm23 expression with anaplastic thyroid carcinoma suggests its correlation with rapid cell proliferation.

The protein product of Nm23 gene has recently been proposed to play an important role in tumour metastasis suppression (Rosengard *et al.*, 1989; Steeg *et al.*, 1989). The Nm23 protein has substantial homology with the protein encoded by a *Drosophila* abnormal wing discs (awd) developmental gene, and nucleoside diphosphate (NDP) kinase, which catalyses the phosphorylation of nucleoside diphosphate into nucleoside triphosphates (Biggs, *et al.*, 1990; Kimura *et al.*, 1990; Wallet *et al.*, 1990). The abundance of Nm23 expression was described to be inversely correlate with metastatic potential in several rodent metastasis model systems: murine k-1735 melanomas (Steeg *et al.*, 1988; Leone *et al.*, 1991), N-nitrosomethylurea-induced rat mammary tumours (Steeg *et al.*, 1988), mouse mammary tumour virus-induced tumours (Steeg *et al.*, 1989) and cotransfected with ras alone or ras plus adenovirus 2E1A rat embryo fibroblasts (Steeg *et al.*, 1988). The expression of human Nm23 gene was also found to be lower in human breast cancer with high metastatic potential than in that with low metastatic potential (Bevilacqua *et al.*, 1989; Barnes *et al.*, 1991; Hennessy *et al.*, 1991; Hirayama *et al.*, 1991). Such a correlation was, however, not observed in some other human tumours such as colon cancer (Haut *et al.*, 1991), neuroblastoma (Hailat *et al.*, 1991), and some solid tumours (Lacombe *et al.*, 1991). The expression of the gene was equally increased in both high and low metastatic colon cancers. In neuroblastoma and some solid tumours Nm23 expression was positively associated with advanced disease stage.

Thyroid carcinoma of follicular origin is one of the most common cancers affecting endocrine tissues. It spans a wide range of biologic behaviour with broadly differing prognoses. In general, differentiated (papillary and follicular) carcinomas grow less aggressively and have a relatively good prognosis, whereas poorly differentiated (anaplastic) carcinomas are very aggressive and most patients die within a year of its diagnosis. The disease course is, however, extremely variable. It is well known that thyroid carcinomas of similar histology can behave divergently in terms of local invasions and distant metastases. Currently there is no reliable way to predict the disease course with confidence. In the present study we examined Nm23 gene expression among different types of thyroid carcinomas to find whether its abundance associated with tumour metastatic behaviour and progression.

### Materials and methods

All tumours were obtained at surgery from King Faisal Specialist Hospital and Research Centre in Saudi Arabia. Tissues were immediately frozen in liquid nitrogen and stored at − 70°C until processed. The clinical staging was based on the TNM classification of thyroid carcinoma (DeGroot & Sridama, 1989), stage I: intrathyroidal involvement only, stage II: regional lymph node involvement, stage III: adjacent tissue invasion, and stage IV: distant metastasis. Five multinodular goitres (adenomatous hyperplasia), one follicular adenoma, 26 papillary carcinomas, three follicular carcinomas and four anaplastic carcinomas were studied.

Full-length human Nm23-H1 cDNA probe was kindly donated by Dr Patricia Steeg of National Cancer Institute, Bethesda, Maryland, USA.

The oligonucleotide probe for 18S ribosomal RNA was synthesised and the sequence is as follows: 5′-GGTCAGCG-CTCGTCGGCATGTAATAG-3′.

### *RNA extraction and Northern hybridisation*

Total RNA was extracted by the guanidinium thiocyanate-phenol-chloroform method (Chomczynski *et al.*, 1987). Twenty μg of total RNA was fractionated on 1% agarose gel containing 2.2 M formaldehyde and blotted onto nylon membranes (Hybond-N, Amersham) by capillary transfer. The accuracy of RNA loading was monitored by ethidium bromide staining and later by hybridisation to an oligoprobe for 18S ribosomal RNA as previously described (Shi *et al.*, 1991). The Nm23-H1 cDNA probe was labelled with [α-$^{32}$P]-dCTP to a specific activity of $10^9$ c.p.m. $\mu g^{-1}$ using Pharmacia's random primer labelling kit. Hybridisation was performed at 42°C for 18 h in 6 × SSPE, 10 mM EDTA, 5 × Denhardt's solution, 0.5% SDS, 100 $\mu g\, ml^{-1}$ denatured salmon testis DNA and 50% formamide. The membranes were then washed twice in 2 × SSPE at 65°C and exposed to Kodak XAR-5 film at − 70°C with intensifying screens.

Following autoradiography band intensities were quantitated by a Bio-Rad scanning laser densitometry and normal-

Correspondence: N.R. Farid, Department of Medicine, MBC 46, King Faisal Specialist Hospital and Research Centre, PO Box 3354, Riyadh 11211, Kingdom of Saudi Arabia.
Received 4 November 1992; and in revised form 17 March 1993.

ised by comparison with the 18S ribosomal band. Comparison between specimen groups was done using the unpaired Student's *t*-test.

*PCR-SSCP analysis*

Five μg of total RNA was reverse transcribed into cDNA in 15 μl volume, using Pharmacia's first-strand cDNA synthesis kit. Four PCR primers were synthesised, two of which (primer 1 and 4) flanked the coding region of the Nm23-H1 gene and the other two (primer 2 and 3) were from the middle of the coding region. Primers 1 and 2 (5'-CAGCCG-GAGTTCAAACCTAA-3', 5'-TTGGTCTCCCCGAGCATG-ACT-3'), and Primers 3 and 4 (5'-GGGCTGAATGTGGTG-AAGACG-3', 5'-GGATGTGAAAAGCAATGTGG-3') were used to generate by PCR two overlapping fragments, each about 350 bp. 0.5 μl of tumour cDNA was used for PCR in 25 μl of buffer containing 0.2 μM primers end-labelled with [γ]-$^{32}$P]ATP. Samples were denatured at 94°C for 3 min and submitted to 25 cycles of amplification under the following conditions: 40 s denaturation at 94°C, 40 s annealing at 56°C, and 40 s extension at 72°C.

Single-strand conformation polymorphism analysis was done as described previously (Orita *et al.*, 1989*a*,*b*). Briefly, 1 μl of each amplified product was diluted in 20 μl loading buffer containing 95% formamide, 20 mM EDTA, 0.05% bromophenol blue and 0.05% xylene cyanol, and heated at 95°C for 3 min. Two μl was loaded into 5% non-denaturing polyacrylamide gel with 10% glycerol and run at room temperature, 30 watts for 6 h. The gel was then exposed to Kodak XAR-5 film overnight at − 70°C.

*DNA sequencing analysis*

DNA sequencing was performed by the dideoxy chain termination method after cloning the PCR products into TA cloning vector (Invitrogen Co., CA, USA).

**Figure 2** Nm23 expression in thyroid tumours. The levels of Nm23 mRNA were quantitated densitometrically from Northern blots and blotted against multinodular goitres and thyroid tumours with different histologic types and clinical stages. The only follicular adenoma specimen was grouped with multinodular goitres. The levels of 18S ribosomal RNA were used as an internal standard to correct variations in the amount of RNA loaded. Means and standard errors of the mean are indicated adjacent to the dot plots.

### Results

The abundance of Nm23 mRNA was examined in tissues from five multinodular goitres and 34 thyroid tumours. Nm23 was expressed in all the thyroid tissue specimens. The expression level was, however, variable in different stages of thyroid carcinoma. Figure 1 shows a representative Northern blot hybridisation. The expression level of Nm23 gene was quantitated by laser densitometry and compared with different stages of thyroid carcinoma. As shown in Figure 2,

**Figure 1** Northern blot hybridisation of Nm23 gene expression in 14 thyroid specimens. Total RNA was electrophoresed on agarose/formaldehyde gel and blotted onto a nylon membrane. Hybridisation was carried out with a full-length Nm23-H1 cDNA probe (upper panel), and an oligoprobe for 18S ribosomal RNA to monitor RNA loading (lower panel). PC: papillary carcinoma; AC: anaplastic carcinoma; Goitre: multinodular goitre; Roman numerals stand for different disease stages.

multinodular goitres have a value of 5.15 ± 0.53 (mean ± SEM); Stage I carcinomas, 6.74 ± 1.30; Stage II carcinomas, 5.61 ± 0.47; Stage III carcinomas, 5.16 ± 0.76; Stage IV carcinomas, 9.23 ± 1.30; Anaplastic carcinomas, 12.85 ± 1.55. Therefore, in early stages of differentiated thyroid carcinoma (stage I and II), Nm23 expression was comparable to that in multinodular goitres. In stage III thyroid carcinomas, Nm23 mRNA was reduced in 50% of specimens. The average level at this stage was, however, not reduced (Figure 2). Surprisingly, in advanced stages of thyroid carcinomas (stage IV and anaplastic), Nm23 gene expression was significantly increased ($P < 0.01$, unpaired *t*-test) (Figures 1 & 2). The tumour specimens were reviewed by an independent pathologist. There was no clear correlation of Nm23 expression with the proportion of stromal tissues present in the tumour samples. It is, therefore, unlikely that stromal tissues contributed significantly to the high intra-group variation of Nm23 expression.

The wide variation of Nm23 gene expression in different stage thyroid tumours may have resulted from mutations or deletions in the coding region of the gene. To rule out such a possibility, we screened for Nm23-H1 gene mutations in these thyroid tumour specimens by RT-PCR-SSCP technique. Primers 1 and 4 were used to specifically amplify Nm23-H1 coding sequence. No mutations were found although we did find p53 mutations in nine of these tumour specimens using the same cDNA mix and technique. Four cDNA samples generated by PCR were randomly chosen and the coding region was sequenced. Two of them were from anaplastic carcinoma specimens which had high level Nm23 expression, whereas the other two were from papillary carcinoma specimens which had reduced Nm23 expression. None of the specimens sequenced was found to have mutations.

### Discussion

The data presented herein demonstrate that Nm23 was expressed in both benign and malignant thyroid lesions. By contrast to the findings reported in some tumours such as breast cancer (Bevilacqua *et al.*, 1989; Barnes *et al.*, 1991; Hennessy *et al.*, 1991; Hirayama *et al.*, 1991) and melanoma (Steeg *et al.*, 1988; Leone *et al.*, 1991) that high metastatic potential correlates with lower Nm23 expression, our results show that the expression of Nm23 is further increased in advanced stage of thyroid carcinomas. The mechanism for this increase and its effect on the tumour progression is not clear. It is likely that in certain tumours such as neuroblastoma (Hailat *et al.*, 1991), colon cancer (Haut *et al.*, 1991) and thyroid tumour (this study), Nm23 expression reflects cell proliferation. This conclusion is supported by recent study showing Nm23-H1 expression is related to cell proliferation (Keim *et al.*, 1992) and our unpublished observation that Nm23 was expressed at higher levels in the early stage as compared to the late stage of mouse embryogenesis.

It is known that Nm23 gene product has NDP kinase activity which provides intracellular pools of nucleoside triphosphates (excluding ATP) required for nucleic acid synthesis (Liotta & Steeg, 1990; Gilles *et al.*, 1991). In many systems, NDP kinases have been found associated with GTP binding proteins including elongation factor (Walton & Gill, 1975; Ohtsuki & Yokoyama, 1987), microtubules (Nickerson & Wells, 1984) and p21 (Ohtsuki *et al.*, 1986) or Gs$\alpha$ (Kimura & Shimada, 1988; Otero, 1990), suggesting that they are involved in processes like protein synthesis, tubulin polymerisation in the mitotic spindle and cytoskeleton, and signal transduction by supplying GTP to GTP binding proteins. Thus a high level Nm23 expression could possibly induce pleiotropic effects on cellular functions.

A second human Nm23 gene (Nm23-H2) has been discovered recently. It encodes a protein with a predicted Mr = 17,000 and is 88% identical to the Nm23-H1 protein sequence (Stahl *et al.*, 1991). The Nm23-H1 probe we used does not efficiently distinguish between the two Nm23 mRNAs. Northern blot hybridisation of Nm23-H2-specific probe to breast tumours and cell lines shows that Nm23-H2 expression was also reduced in high metastatic potential tumour cells but to a less extent than Nm23-H1 (Stahl *et al.*, 1991), indicating that the two genes are regulated independently. It is thus possible that the observed Nm23 expression in thyroid tumours could be one of several distinct forms of Nm23 that are variably expressed in different cell types and that could play different roles.

Somatic allelic deletions of Nm23-H1 (including homozygous deletions in colon cancer) have been reported in human cancers such as breast, kidney, colon and lung (Cohn *et al.*, 1991; Leone *et al.*, 1991). Cohn *et al.* (1991) demonstrated that 73% patients with Nm23-H1 deletions in colon cancer had distant metastases as compared to 20% without Nm23-H1 deletions, supporting its anti-metastatic role in colon cancers. Their studies seem to contradict the report by Haut *et al.*, who found that Nm23 mRNA levels were higher in invasive colon cancers (Haut *et al.*, 1991). Further studies are required to prove that the increased Nm23 product is not mutant forms. In our study, we have shown that no mutations were detected in the coding region of Nm23-H1 by SSCP technique. However, we cannot completely exclude the possibility of mutation, especially in the regulatory region of Nm23-H1. Moreover, we do not yet know whether allelic deletions of Nm23-H1 or mutations of Nm23-H2 exist in thyroid tumours.

In summary, Nm23 is expressed in both benign and malignant thyroid lesions. No correlation could be demonstrated between high level Nm23 expression and its anti-metastatic potential. Therefore, Nm23 mRNA level cannot be used as a marker of low metastatic potential in thyroid carcinomas. The high level Nm23 expression in advanced thyroid carcinoma suggests that it may be participated in cell proliferation. Tissue-specific factors may be involved in the dissociation of Nm23 expression from its anti-metastatic activity.

We wish to thank Dr Patricia Steeg for the generous gift of Nm23 cDNA, and Drs A.M. Aktar, M. Ali, S. Ingemansson, S.S. Hussain and A. Nasim for helping us secure the specimens for analysis in this study. Oligonucleotide synthesis was done by Mr Philip Ahring.

### References

BARNES, R., MASOOD, S., BARKER, E., ROSENGARD, A.M., COGGIN, D.L., CROWELL, T., KING, C.R., PORTER-JORDAN, K., WARGOTZ, E.S., LIOTTA, L.A. & STEEG, P.S. (1991). Low nm23 protein expression in infiltrating ductal breast carcinomas correlates with reduced patient survival. *Am. J. Pathol.*, **139**, 245–250.

BEVILACQUA, G., SOBEL, M.E., LIOTTA, L.A. & STEEG, P.S. (1989). Association of low nm23 RNA levels in human primary infiltrating ductal breast carcinomas with lymph node involvement and other histopathological indicators of high metastatic potential. *Cancer Res.*, **49**, 5185–5190.

BIGGS, J., HERSPERGER, E., STEEG, P.S., LIOTTA, L.A. & SHEARN, A. (1990). A *Drosophila* gene that is homologous to a mammalian gene associated with tumor metastasis codes for a nucleoside diphosphate kinase. *Cell*, **63**, 933–940.

CHOMCZYNSKI, P. & SACCHI, N. (1987). Single-step method of RNA isolation by acid guanidinium thiocyanate-phenol-chloroform extraction. *Anal. Biochem.*, **162**, 156–159.

COHN, K.H., WANG, F., DESOTO-LAPAIX, F., SOLOMON, W.B., PATTERSON, L.G., AMOLD, M.R., WEIMAR, J., FELDMAN, J.G., LEVY, A.T., LEONE, A. & STEEG, P.S. (1991). Association of nm23-H1 allelic deletions with distant metastases in colorectal carcinoma. *Lancet*, **338**, 722–724.

DEGROOT, L.J. & SRIDAMA, V. (1989). Thyroid neoplasia. In DeGroot, L.J. (eds), *Endocrinology*, **2**, pp. 758–776. Philadelphia: W.B. Saunders Co.

GILLES, A.-M., PRESECAN, E., VONICA, A. & LASCU, I. (1991). Nucleoside diphosphate kinase from human erythrocytes. *J. Biol. Chem.*, **266**, 8784–8789.

HAILAT, N., KEIM, D.R., MELHEM, R.F., ZHU, X.-X., ECKERSKORN, C., BRODEUR, G.M., REYNOLDS, C.P., SEEGER, R.C., LOTTSPEICH, F., STRAHLER, J.R. & HANASH, S.M. (1991). High levels of p19/nm23 protein in neuroblastoma are associated with advanced stage disease and with N-*myc* gene amplification. *J. Clin. Invest.*, **88**, 341–345.

HAUT, M., STEEG, P.S., WILLSON, J.K.V. & MARKOWITZ, S.D. (1991). Induction of nm23 gene expression in human colonic neoplasms and equal expression in colon tumors of high and low metastatic potential. *J. Natl Cancer Inst.*, **83**, 712–716.

HENNESSY, C., HENRY, J.A., MAY, F.E., WESTLEY, B.R., ANGUS, B. & LENNARD, T.W. (1991). Expression of the antimetastatic gene nm23 in human breast cancer: an association with good prognosis. *J. Natl Cancer Inst.*, **83**, 281–285.

HIRAYAMA, R., SAWAI, S., TAKAGI, Y., MISHIMA, Y., KIMURA, N., SHIMADA, N., ESAKI, Y., KURASHIMA, C., UTSUYAMA, M. & HIROKAWA, K. (1991). Positive relationship between expression of anti-metastatic factor (nm23 gene product or nucleoside diphosphate kinase) and good prognosis in human breast cancer. *J. Natl Cancer Inst.*, **83**, 1249–1250.

KEIM, D., HAILAT, N., MELHEM, R., ZHU, X.X., LASCU, I., VERON, M., STRAHLER, J. & HANASH, S.M. (1992). Proliferation-related expression of p19/nm23 nucleoside diphosphate kinase. *J. Clin. Invest.*, **89**, 919–924.

KIMURA, N. & SHIMADA, N. (1988). Membrane associated nucleoside diphosphate kinase from rat liver. *J. Biol. Chem.*, **263**, 4647–4653.

KIMURA, N., SHIMADA, N., NOMURA, K. & WATANABE, K. (1990). Isolation and characterization of a cDNA clone encoding rat nucleoside diphosphate kinase. *J. Biol. Chem.*, **265**, 15744–15749.

LACOMBE, M.-L., SASTRE-GARAU, X., LASCU, I., VONICA, A., WALLET, V., THIERY, J.P. & VERON, M. (1991). Overexpression of nucleoside diphosphate kinase (nm23) in solid tumors. *Eur. J. Cancer*, **27**, 1302–1307.

LEONE, A., FLATOW, U., KING, C.R., SANDEEN, M.A., MARGULIES, I.M.K., LIOTTA, L.A. & STEEG, P.S. (1991). Reduced tumor incidence, metastatic potential, and cytokine responsiveness of nm23 transfected melanoma cells. *Cell*, **65**, 25–35.

LEONE, A., McBRIDE, O.W., WESTON, A., WANG, M.G., ANGLARD, P., CROPP, C.S., GOEPEL, J.R., LIDEREAU, R., CALLAHAN, R., LINEHAN, W.M., REES, R.C., HARRIS, C.C., LIOTTA, L.A. & STEEG, P.S. (1991). Somatic allelic deletion of nm23 in human cancer. *Cancer Res.*, **51**, 2490–2493.

LIOTTA, L.A. & STEEG, P.S. (1990). Clues to the function of Nm23 and Awd proteins in development, signal transduction and tumor metastasis provided by studies of *Dictyostelium discoideum*. *J. Natl Cancer Inst.*, **82**, 1170–1173.

NICKERSON, J.A. & WELLS, W.W. (1984). The microtubule-associated nucleoside diphosphate kinase. *J. Biol. Chem.*, **259**, 11297–11304.

OHTSUKI, K., IKEUCHI, T. & YOKOYAMA, M. (1986). Characterization of nucleoside diphosphate kinase-associated guanine nucleotide-binding proteins from Hela S3 cells. *Biochim. Biophys. Acta.*, **882**, 322–330.

OHTSUKI, K. & YOKOYAMA, M. (1987). Direct activation of guanine nucleotide binding proteins through a high-energy phosphate-transfer by nucleoside diphosphate-kinase. *Biochem. Biophys. Res. Commun.*, **148**, 300–307.

ORITA, M., IWAHANA, H., KANAZAWA, H., HAYASHI, K. & SEKIYA, T. (1989*a*). Detection of polymorphisms of human DNA by gel electrophoresis as single-strand conformation polymorphisms. *Proc. Natl Acad. Sci. USA*, **86**, 2766–2770.

ORITA, M., SUZUKI, Y., SEKIYA, T. & HAYASHI, K. (1989*b*). Rapid and sensitive detection of point mutations and DNA polymorphisms using the polymerase chain reaction. *Genomics*, **5**, 874–879.

OTERO, A.D. (1990). Transphosphorylation and G protein activation. *Biochem. Pharmacol.*, **39**, 1399–1404.

ROSENGARD, A.M., KRUTZSCH, H.C., SHEARN, A., BIGGS, J.R., BARKER, E., MARGUILES, I.M.K., KING, C.R., LIOTTA, L.A. & STEEG, P.S. (1989). Reduced Nm23/Awd protein in tumor metastasis and aberrant *Drosophila* development. *Nature*, **342**, 177–180.

SHI, Y., ZOU, M., SCHMIDT, H., JUHASZ, F., STENSKY, V., ROBB, D. & FARID, N.R. (1991). High rates of *ras* codon 61 mutation in thyroid tumors in an iodide-deficient area. *Cancer Res.*, **51**, 2690–2693.

STAHL, J.A., LEONE, A., ROSENGARD, A.M., PORTER, L., KING, C.R. & STEEG, P.S. (1991). Identification of a second human nm23 gene, nm23-H2. *Cancer Res.*, **51**, 445–449.

STEEG, P.S., BEVILACQUA, G., KOPPER, L., THORGEIRSSON, U.P., TALMADGE, J.E., LIOTTA, L.A. & SOBEL, M.E. (1988). Evidence for a novel gene associated with low tumor metastatic potential. *J. Natl Cancer Inst.*, **80**, 200–204.

STEEG, P.S., BEVILACQUA, G., POZZATTI, R., LIOTTA, L.A. & SOBEL, M.E. (1988). Altered expression of nm23, a gene associated with low tumor metastatic potential, during adenovirus 2 Ela inhibition of experimental metastasis. *Cancer Res.*, **48**, 6550–6554.

STEEG, P.S., BEVILACQUA, G., ROSENGARD, A.M., SOBEL, M.E., CIOCE, V. & LIOTTA, L.A. (1989). Altered gene expression in tumor metastasis: the nm23 gene. In Schirrmacher, V. & Schwartz-Albiez, R. (eds), *Cancer Metastasis. Molecular and Cellular Biology, Host Immune Responses and Perspectives for Treatment*, pp. 48–52. Berlin: Springer Verlag.

WALLET, V., MUTZEL, R., TROLL, H., BARZU, O., WURSTER, B., VERON, M. & LACOMBE, M.-L. (1990). *Dictyostelium* nucleotide diphosphate kinase highly homologous to nm23 and awd proteins involved in mammalian tumor metastasis and *Drosophila* development. *J. Natl Cancer Inst.*, **82**, 1199–1202.

WALTON, G. & GILL, G. (1975). Nucleotide regulation of a eukaryotic protein synthesis initiation complex. *Biochim. Biophys. Acta.*, **390**, 231–245.